# Reverse sural flap for ankle and heel soft tissues reconstruction

**Published:** 2017

**Authors:** RN Ciofu, DG Zamfirescu, SA Popescu, I Lascar

**Affiliations:** * “Sf. Maria” Hospital, Bucharest, Romania; ** “Zetta” Clinic, Bucharest, Romania; *** Bucharest Clinical Emergency Hospital, Bucharest, Romania; “Carol Davila” University of Medicine and Pharmacy, Bucharest, Romania

**Keywords:** reverse sural flap, ankle, heel, soft tissues reconstruction

## Abstract

**Introduction:** The potential of the medial calf integument, as donor site for a free flap based on musculocutaneous branches of the medial sural artery, was first identified by Taylor and Daniel, following cadaver investigation. In 1981, Pontén described the fasciocutaneous sural flap as a reconstructive option for soft tissue loss of the lower extremity, particularly around the knee. Two years later, Donski and Fogdestram presented the distally based fasciocutaneous flap from the sural region followed by Montegut and Allen who considered the sural artery perforator flap as a viable alternative for the gastrocnemius myocutaneous flap.

The sural flap proved a considerable versatility at the level of the lower leg (from the knee to the ankle and heel) as well as for other anatomical regions. The most common usage of the flap is for the distal-third defects of the leg.

**Materials and method:** A group of 10 patients with soft tissue losses at the ankle or heal due to a various etiopathogeny represented by cancer excision, trauma, unstable scars, chronic osteomyelitis, in which a microsurgical free transfer had no indication or was not wanted, was presented.

Our group reported a 30% complication rate in a high-risk patient population, including patients with diabetes mellitus, peripheral vascular disease, and venous insufficiency.

**Results:** All the defects were covered successfully, without major complications. Usually, only a minor margin of the tip of the flap was lost, which was easily solved with a guided secondary healing. Most flaps showed a slight venous congestion, which cleared in a few days.

The functional result was very good in all the patients, while the aesthetic appearance was acceptable even in female patients.

**Discussion:** An ideal indication of a reverse sural flap may be a defect over an intact but partially exposed Achilles tendon.

**Conclusions:** The sural reverse flap is useful in the ankle and foot soft tissues reconstruction whenever we have reasons not to use a microsurgical free transfer.

Venous congestion with consecutive partial or complete flap loss is a common complication, so this would not be recommended in patients with obvious acute or chronic venous stasis.

The reverse sural island flap should no longer be regarded as a flap of secondary choice to free tissue transfer, but as an equally valuable alternative for small and midsized defects around the ankle and heel.

## Introduction

The potential of the medial calf integument, as donor site for a free flap based on musculocutaneous branches of the medial sural artery, was first identified by Taylor and Daniel, following cadaver investigation [**1**]. In 1981, Pontén [**2**] described the fasciocutaneous sural flap as a reconstructive option for soft tissue loss of the lower extremity, particularly around the knee. Two years later, Donski and Fogdestram [**3**] presented the distally based fasciocutaneous flap from the sural region followed by Montegut and Allen who considered the sural artery perforator flap as a viable alternative for the gastrocnemius myocutaneous flap [**4**]. In 1992, Masquelet et al. introduced the concept of neurocutaneous island flap and described the sural neurocutaneous flap [**5**], lately referred to as retrograde sural nerve flap [**6**]. Hallock and Cavadas further investigated the vascular anatomy of the sural perforator flap and reported a series of clinical cases successfully managed by using it [**7**-**9**]. 

In the last decades, multiple modifications of the sural flap have been reported and a plethora of often puzzling denominations appeared. According to various classification criteria, but mostly determined by the surgical technique for harvesting and using this flap in various defects reconstruction, the sural flap has been referred to as reverse sural artery flap [**10**,**11**], delayed sural flap [**12**], supercharged reverse sural flap [**13**], sural fasciomusculocutaneous flap [**14**], distally based sural flap [**15**,**16**], cross-leg distally based sural flap [**17**], distally based sural neurocutaneous flap [**18**], distally based sural neuro-fasciomyocutaneous flap [**19**], distally based sural neuro-lesser saphenous veno-fasciocutaneous compound flap [**20**], nerve sparing distally based sural fasciocutaneous flap [**21**], etc.

Summarizing, one can state that what is generally meant by “sural flap” is a variant of the reverse sural flap or distally based sural flap [**22**] (the word “sural” describing the sural angiosome, meaning the site where the flap’s skin island is harvested). 

## Surgical anatomy

The two sural branches of the peroneal artery and the posterior tibial artery give the main arterial supply of the medial calf. The medial and lateral sural arteries arise from the popliteal artery superior to the tibiofemoral join. In some cases, the popliteal artery gives one common sural artery that subsequently bifurcates into medial and lateral branches. Rarely, none or more than one medial sural artery has been identified [**15**,**23**]. The medial superficial sural artery courses above the fascia (sometimes accompanying the medial sural cutaneous nerve) before penetrating the fascia at the mid calf level [**6**]. At the level of the tibiofemoral join line, the sural arteries, paired with a motor branch of the tibial nerve, enter the deep surface of medial and lateral heads of the gastrocnemius muscle. Their cutaneous branches (medial and lateral superficial sural arteries) supply the skin of the posterior leg [**24**]. The lesser saphenous vein provides the venous drainage of the region. Between the gastrocnemius heads, it courses along the midline of the calf accompanying the median sural artery between the two gastrocnemius heads, penetrates the fascia and enters the popliteal vein in the mid popliteal fossa. A plexus of small vessels is seen around the medial sural nerve (which innervates the calf) and the lesser saphenous vein [**5**,**6**,**16**,**20**]. Superficially, the sural nerve, the sural artery (arteries) and the lesser saphenous are the structures supplying the sural flap. 

The perforating vessels in the calf region mainly originate from medial sural arteries and peroneal artery. The medial sural artery gives at least one large perforator while the lateral sural artery perforators are either inconsistent in location or absent [**7**,**25**]. On average, there are 4 musculo-fasciocutaneous perforators, of 0.2-0.5 mm in diameter, between the suprafascial sural neurovascular axis and the deep gastrocnemius muscle [**26**]. The perforators tend to course obliquely after leaving the heads of gastrocnemius, before piercing the deep fascia [**7**]. When the superficial sural vessels are greater than 1mm in diameter, the musculocutaneous perforators are insignificant. 

The results of the anatomical study on 20 lower limbs of human cadavers carried out by the authors of the present article [**23**], showed an average of 1.9 muscular perforating vessels with an outer diameter between 0.4-1.1 mm originating from the medial sural artery. Each perforator presented a comittant vein of 0.6-1.1 mm in diameter. The perforant caliber proved to be inversely proportional to the number of vessels: in the specimen containing 5 perforant vessels, the caliber varied from 0.4 to 0.6 mm while in those presenting 1-2 perforants, the diameter was of 0.9-1.1 mm. The closer to the line between the popliteal fold and the medial malleolus the perforants are situated, the larger both the diameter and the length of the vascular pedicle are. 90% of the perforators have a pedicle that is large enough to facilitate any requisite microanastomoses. All these results are in line with the conclusions of the previous studies developed internationally [**7**-**9**,**25**-**28**]. 

## Design and surgical technique

Located over the midline raphe between the two heads of the gastrocnemius muscle and between the popliteal fossa and the mid portion of the leg, the sural flap was described by Cormack and Lamberty as one of the longest fasciocutaneous flap of the leg [**29**]. Based on peroneal perforators, the flap can safely extend three quarters of the leg proximally [**30**]. Flap planning implies the thorough identification and selection of perforators overlying the lateral malleolus [**7**]. The Doppler probe is most useful in identifying the perforators. 

For flap dissection, the patient is positioned in the prone position. The flap is marked on the skin in the form of an ellipse centered on the raphe between the two gastrocnemius muscle bodies, whose projection is visible on the posterior aspect of the leg. The incision starts on the lateral and superior borders of the flap and continues in the subfascial plane until the sural nerve is identified in the median raphe. Then the incision goes on the other boundaries of the flap and the subfascial dissection continues with the ligation of all the perforators from the gastrocnemius belly and the inclusion of the septum between the muscles in the flap. The sural nerve is attached to the fascia at the superior border of the flap.

## Clinical applications 

The sural flap proved a considerable versatility at the level of the lower leg (from the knee to the ankle and heel) as well as for other anatomical regions. The most common usage of the flap is for the distal-third defects of the leg. Here, the reverse sural flap permits the soft tissue reconstruction without the need for microsurgery. It does not sacrifice any of the three major arteries to the distal extremity. The flap has a variety of indications for difficult defects even in the most obese or vascularly compromised patients [**24**,**31**,**32**]. Sparing the major artery, the relatively simple dissection and the low donor site morbidity are among the most important advantages of using this flap. 

On the other hand, there are some critical aspects to be considered. The most important one concerns the venous congestions and flap ischemia. Flap delay [**12**,**15**,**30**,**33**,**34**], supercharging [**13**], harvesting the pedicle with 3 cm of tissue on either side and with the overlying skin intact [**35**] are some procedures that help overcoming this complication. Most authors mention the unaesthetic scar at the donor site, mainly if the closure needs a skin graft. Because the flap is harvested with the nerve, the loss of sensibility on the lateral aspect of the foot might pose certain problems [**6**].

## Materials and methods

A group of 10 patients with soft tissue losses at the ankle or heal due to a various etiopathogeny represented by cancer excision, trauma, unstable scars, chronic osteomyelitis, in which a microsurgical free transfer had no indication or was not wanted, was presented.

## Results

Our group reported a 30% complication rate in a high-risk patient population, including patients with diabetes mellitus, peripheral vascular disease, and venous insufficiency.

All the defects were covered successfully, without major complications. Usually, only a minor margin of the tip of the flap was lost, which was easily solved with a guided secondary healing. Most flaps showed a slight venous congestion, which cleared in a few days. In two patients, a delayed flap procedure was used and a positive impact was proved on the flap viability. There were no complaints related to the sacrifice of the sural nerve – the paresthesia on the lateral border of the foot did not create major problems and disappeared within two months. The functional result was very good in all the patients, while the aesthetic appearance was acceptable even in female patients. 

**Fig. 1:**
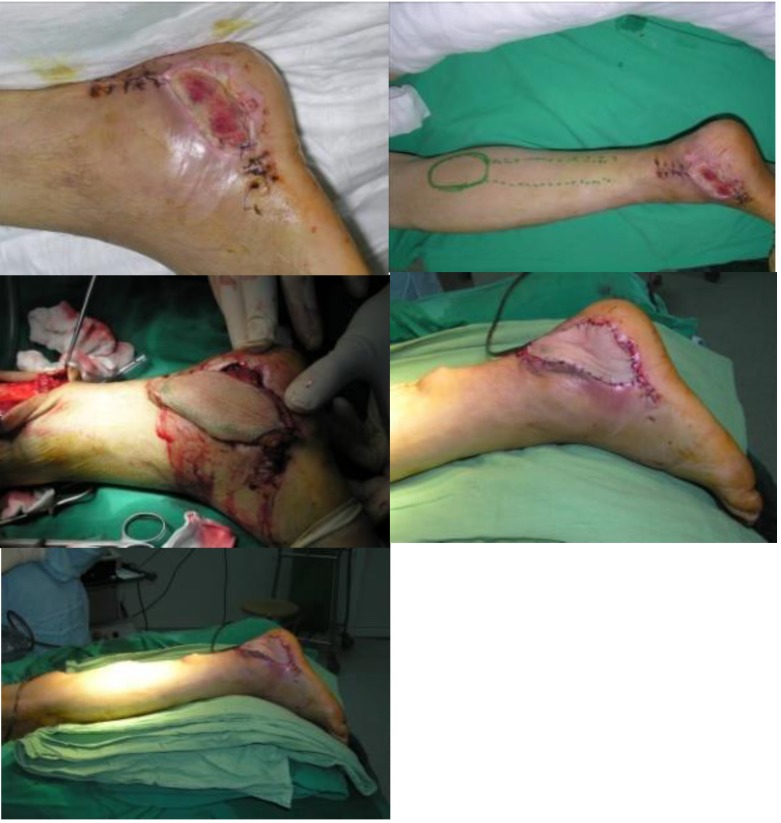
Sural flap for ankle soft tissue defect reconstruction

**Fig. 2:**
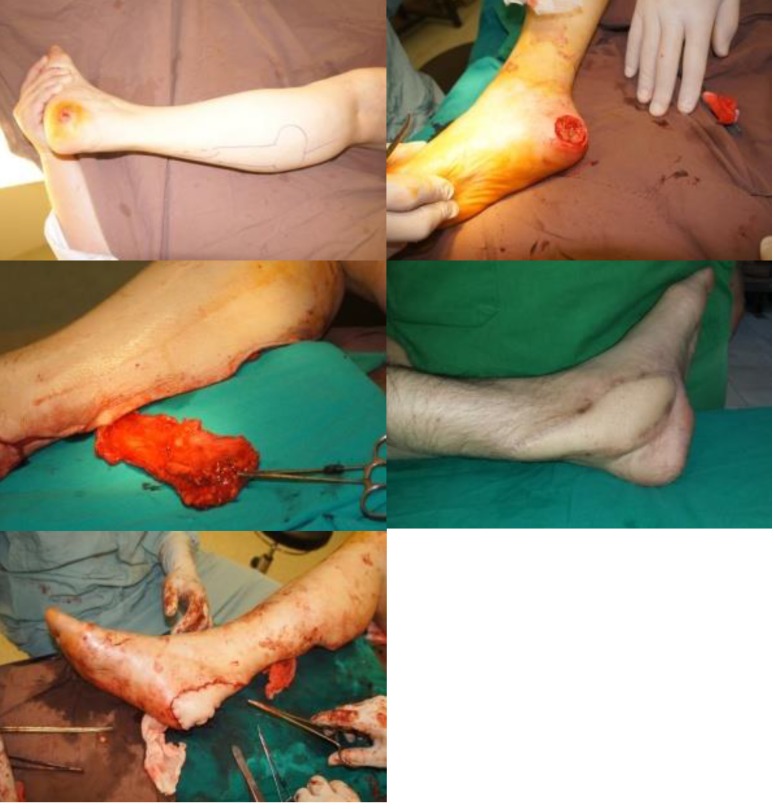
Sural flap for heel soft tissue defect reconstruction

**Fig. 3:**
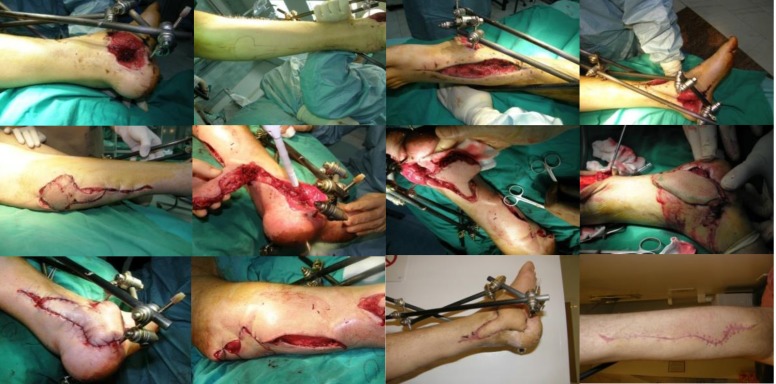
Delayed reverse sural flap for ankle soft tissue defect after tumor excision

**Fig. 4:**
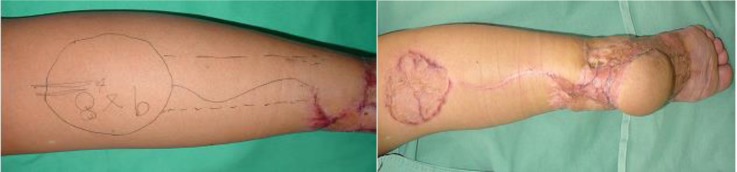
Delayed reverse sural flap for heel reconstruction (case 2)

## Discussions

Venous congestion with partial or complete flap loss is the most feared complication. However, similar risk factors may lead to higher complication rates in lower extremity reconstruction with free tissue transfer as well. Leg elevation, insertion of a small intravenous catheter in the proximal stump of the lesser saphenous vein, or venous supercharging may overcome this problem.

A reverse sural flap delay procedure has been recommended by some authors to prevent flap complications [**12**,**15**,**30**,**33**,**34**]. A delay implies redirecting the blood flow either by transecting the vessel or by incising the lateral edges of the skin island.

An ideal indication of a reverse sural flap may be a defect over an intact but partially exposed Achilles tendon.

## Conclusions

The sural reverse flap is useful in the ankle and foot soft tissues reconstruction whenever we have reasons not to use a microsurgical free transfer. Drawbacks of this flap are the venous congestion, the volume of the flap, which is sometimes not suited for the reconstructed area, and thus the aesthetic appearance, and an additional unsightly donor site defect, but the mechanic properties of the integrated flap are very good.

Venous congestion with consecutive partial or complete flap loss is a common complication, so this would not be recommended in patients with obvious acute or chronic venous stasis.

The reverse sural island flap should no longer be regarded as a flap of secondary choice to free tissue transfer, but as an equally valuable alternative for small and midsized defects around the ankle and heel.
